# Scientific Community Image Forum: A discussion forum for scientific image software

**DOI:** 10.1371/journal.pbio.3000340

**Published:** 2019-06-19

**Authors:** Curtis T. Rueden, Jeanelle Ackerman, Ellen T. Arena, Jan Eglinger, Beth A. Cimini, Allen Goodman, Anne E. Carpenter, Kevin W. Eliceiri

**Affiliations:** 1 Laboratory for Optical and Computational Instrumentation, University of Wisconsin at Madison, Madison, Wisconsin, United States of America; 2 Imaging Platform, Broad Institute of Harvard and MIT, Cambridge, Massachusetts, United States of America; 3 Facility for Advanced Imaging and Microscopy, Friedrich Miescher Institute for Biomedical Research, Basel, Switzerland

## Abstract

Forums and email lists play a major role in assisting scientists in using software. Previously, each open-source bioimaging software package had its own distinct forum or email list. Although each provided access to experts from various software teams, this fragmentation resulted in many scientists not knowing where to begin with their projects. Thus, the scientific imaging community lacked a central platform where solutions could be discussed in an open, software-independent manner. In response, we introduce the Scientific Community Image Forum, where users can pose software-related questions about digital image analysis, acquisition, and data management.

## Abstract 2

Forums and email lists play a major role in assisting scientists in using software, but the existence of multiple forums can result in fragmented communities. This Community Page article introduces the Scientific Community Imaging Forum, where users can pose both software-specific questions, as well as more general questions about digital image acquisition, analysis, and data management.

Imaging is a vital part of modern science. The imaging sciences cross many research fields and modalities and require a wide spectrum of analysis tools and techniques. Although this vast array of analytic options makes imaging a powerful tool, the expanse of choices can also be overwhelming. There is tremendous potential for researchers to lean on each other’s expertise and insight, especially when dealing with a seemingly endless list of image analysis tools and workflows that keeps growing and changing from day to day.

To catalyze better communication about image analysis tools, the ImageJ and CellProfiler open-source imaging software teams recently joined forces to develop the Scientific Community Image Forum (https://forum.image.sc/). The rationale is to facilitate unification and visibility of discussions across the imaging community. Its mission is to ease and document communications across the image analysis community. The focus of this forum is software-oriented aspects of scientific imaging, particularly (but not limited to) image analysis, processing, acquisition, storage, and management of digital scientific images. The primary objective is to foster independent learning for researchers everywhere while maximally leveraging experts’ time by making their guidance available to everyone.

A large collection of community partners have joined the forum, which began as a merging of existing posts in the separate ImageJ and CellProfiler Discourse forums (https://www.discourse.org/), yielding approximately 10,000 users in total and more than 15,000 posts per year. A community partner is an open-source software project or other community organization that uses the Scientific Community Image Forum as a primary discussion channel. This means that (1) the organization links to the forum in its documentation regarding how users should seek support and/or discussion; and (2) it does not promote additional, separate, project-specific discussion channels more prominently than this forum. Each community partner appears in the top navigation with its logo and link to its associated tag feed. Thus far, community partners include Bio-Formats (https://www.openmicroscopy.org/bio-formats/), BoneJ (http://bonej.org/), CellProfiler (http://cellprofiler.org/), Cytomine (https://cytomine.coop/), DeepLabCut (https://github.com/AlexEMG/DeepLabCut), Fiji (https://imagej.net/Fiji), ilastik (http://ilastik.org/), ImageJ (https://imagej.net/), ImagePy (https://github.com/Image-Py/imagepy), ImgLib2 (https://imagej.net/ImgLib2), ImJoy (https://imjoy.io/), Microscopy Image Browser (MIB) (http://mib.helsinki.fi/), Network of European BioImage Analysts (NEUBIAS) (http://eubias.org/NEUBIAS/), OpenSPIM (https://openspim.org/), SCIFIO (https://scif.io/), scikit-image (https://scikit-image.org/), and SLIM Curve (https://imagej.net/SLIM_Curve). These are open-source software tools, with the exception of NEUBIAS, which is a consortium of bioimage analysts and image analysis developers from 36 European countries and a number of external partners including the United States and Australia. Participation of these NEUBIAS members provides the community with critical access to additional image analysis experts, many of them working as image analysis staff in core facilities across Europe. We welcome additional Community Partners.

Discussion of any and all scientific image software problems and tools are within the forum's scope. This inclusivity is a direct reflection of the strong collaborative approach long seen in the bioimaging community. Rather than compete, many of the bioimage analysis software teams have a long history of collaboration and direct code sharing. The Scientific Community Image Forum is the product of that collaborative model and aims to continue increasing intertool collaboration. As new users ask more questions, and as we expand our list of community partners, the forum will continue to be promoted as a primary discussion channel via individual project websites, documentation sources, and community engagement at conferences and workshops. We aim to reach a broad audience of open-source software users and developers and new community partners.

Posts to the forum can fall under a number of categories, including “Image Analysis,” for questions about image processing and analysis; “Usage & Issues,” for discussing technical questions and problems with scientific image software; “Development,” for programming and development questions about scientific image software; “Websites,” for discussion of websites of all community partners; “Announcements,” for sharing new software releases and upcoming community events; “Job Opportunities,” for posting job positions relating to the field of scientific imaging; and more. Forum threads also can and ideally should be tagged for ease of topic search and navigation; tags include not only the names of software tools used (as mentioned previously for community partners) but also more specific topics such as “plugin,” “macro,” “segmentation,” region of interest (“ROI”), etc. A major benefit of such tagging is cross-software discussion, such as questions regarding the integration of multiple software tools.

The Scientific Community Image Forum embraces the diversity of the scientific imaging community while enabling people to ask, How do I do X? without prior knowledge of any software programs or image analysis pipelines. It is a place where individuals can post questions, along with images and/or code snippets, to aid them in their image analysis workflows or development schemes. The forum gives users access to a wide breadth of experts on various software packages and gives those experts a place to have detailed discussions about specific elements of the software, leading to improved understanding and practical links across toolkits. This is a space where software developers can be directly exposed to the needs of users, facilitating novel feature developments and critical usability improvements.

This new forum’s ultimate goals are to accelerate research, educate scientists in image analysis, encourage open science and reproducible research, and catalyze the creation and improvement of open-source tools and their interoperability. The improved cross-visibility of software packages will also promote their continued survival and development by attracting new users and even collaborations with other developers. In this way, together as an imaging community, we will promote new skill acquisition and subsequent dissemination of validated image analysis tools and tips.

## Abbreviations

MIB: Microscopy Image Browser
NEUBIAS: Network of European BioImage Analysts
ROI: region of interest

